# ERas and COLorectal endoscopic surgery: an Italian society for endoscopic surgery and new technologies (SICE) national report

**DOI:** 10.1007/s00464-022-09212-y

**Published:** 2022-05-02

**Authors:** Marco Milone, Ugo Elmore, Michele Manigrasso, Monica Ortenzi, Emanuele Botteri, Alberto Arezzo, Gianfranco Silecchia, Mario Guerrieri, Giovanni Domenico De Palma, Ferdinando Agresta, ERCOLE Study Group, Ferdinando Agresta, Francesco Pizza, Dario D’Antonio, Francesco Amalfitano, Francesco Selvaggi, Guido Sciaudone, Lucio Selvaggi, Daniela Prando, Fabio Cavallo, Mario Guerrieri, Monica Ortenzi, Giovanni Lezoche, Diego Cuccurullo, Ernesto Tartaglia, Carlo Sagnelli, Andrea Coratti, Angela Tribuzi, Michele Di Marino, Gabriele Anania, Cristina Bombardini, Mauro Pietro Zago, Fulvio Tagliabue, Morena Burati, Salomone Di Saverio, Samuele Colombo, Sara El Adla, Maurizio De Luca, Monica Zese, Dario Parini, Paolo Prosperi, Giovanni Alemanno, Jacopo Martellucci, Stefano Olmi, Alberto Oldani, Matteo Uccelli, Dario Bono, Donatella Scaglione, Roberto Saracco, Mauro Podda, Adolfo Pisanu, Valentina Murzi, Antonino Agrusa, Salvatore Buscemi, Irnerio Angelo Muttillo, Biagio Picardi, Edoardo Maria Muttillo, Leonardo Solaini, Davide Cavaliere, Giorgio Ercolani, Francesco Corcione, Roberto Peltrini, Umberto Bracale, Andrea Lucchi, Laura Vittori, Michele Grassia, Alberto Porcu, Teresa Perra, Claudio Feo, Pierluigi Angelini, Domenico Izzo, Luigi Ricciardelli, Mario Trompetto, Gaetano Gallo, Alberto Realis Luc, Andrea Muratore, Marcello Calabrò, Bruno Cuzzola, Andrea Barberis, Federico Costanzo, Giulio Angelini, Graziano Ceccarelli, Fabio Rondelli, Michele De Rosa, Elisa Cassinotti, Luigi Boni, Ludovica Baldari, Paolo Pietro Bianchi, Giampaolo Formisano, Giuseppe Giuliani, Andrea Alessandro Pisani Ceretti, Nicolò Maria Mariani, Marco Giovenzana, Roberto Farfaglia, Paolo Marcianò, Valeria Arizzi, Micaela Piccoli, Francesca Pecchini, Gianmaria Casoni Pattacini, Emanuele Botteri, Nereo Vettoretto, Claudio Guarnieri, Letizia Laface, Emmanuele Abate, Massimiliano Casati, Carlo Feo, Nicolò Fabri, Antonio Pesce, Piero Maida, Giampaolo Marte, Roberta Abete, Lorenzo Casali, Alessandro Marchignoli, Matteo Dall’Aglio, Stefano Scabini, Davide Pertile, Alessandra Aprile, Jacopo Andreuccetti, Alberto Di Leo, Lorenzo Crepaz, Francesco Maione, Sara Vertaldi, Alessia Chini, Riccardo Rosati, Francesco Puccetti, Giulia Maggi, Andrea Cossu, Alberto Sartori, Maurizio De Luca, Giacomo Piatto, Nicola Perrotta, Marta Celiento, Marco Scorzelli, Vincenzo Pilone, Salvatore Tramontano, Pietro Calabrese, Raffaele Sechi, Nicola Cillara, Giaime Putzu, Michele Guido Podda, Mauro Montuori, Enrico Pinotti, Giuseppe Sica, Marzia Franceschilli, Bruno Sensi, Maurizio Degiuli, Rossella Reddavid, Lucia Puca, Marco Farsi, Alessio Minuzzo, Elena Gia, Gian Luca Baiocchi, Valerio Ranieri, Andrea Celotti, Francesco Bianco, Sebastiano Grassia, Alessandra Novi

**Affiliations:** 1 Department of Clinical Medicine and Surgery, Federico II” University of Naples, via Pansini 5, 80131 Naples, Italy; 2 Division of Gastrointestinal Surgery, San Raffaele Scientific Institute, Milan, Italy; 3 Department of Advanced Biomedical Sciences, “Federico II” University of Naples, via Pansini 5, Naples, Italy; 4 Department of General Surgery, Università Politecnica Delle Marche, Piazza Roma 22, 60121 Ancona, Italy; 5 General Surgery, ASST Spedali Civili Di Brescia, Montichiari, Italy; 6 Department of Surgical Sciences, University of Torino, Turin, Italy; 7 Department of Medical Surgical Science and Biotechnologies, Faculty Pharmacy and Medicine, Sapienza University of Rome, Rome, Italy; 8 Department of General Surgery, Department of General Surgery, Ulss2 Marca Trevigiana, Vittorio Veneto, TV Italy

**Keywords:** Colorectal, ERAS, Enhanced Recovery, Minimally invasive

## Abstract

**Background:**

Several reports demonstrated a strong association between the level of adherence to the protocol and improved clinical outcomes after surgery. However, it is difficult to obtain full adherence to the protocol into clinical practice and has still not been identified the threshold beyond which improved functional results can be reached.

**Methods:**

The ERCOLE (ERas and COLorectal Endoscopic surgery) study was as a cohort, prospective, multi-centre national study evaluating the association between adherence to ERAS items and clinical outcomes after minimally invasive colorectal surgery. The primary endpoint was to associate the percentage of ERAS adherence to functional recovery after minimally invasive colorectal cancer surgery. The secondary endpoints of the study was to validate safety of the ERAS programme evaluating complications’ occurrence according to Clavien-Dindo classification and to evaluate the compliance of the Italian surgeons to each ERAS item.

**Results:**

1138 patients were included. Adherence to the ERAS protocol was full only in 101 patients (8.9%), > 75% of the ERAS items in 736 (64.7%) and > 50% in 1127 (99%). Adherence to > 75% was associated with a better functional recovery with 90.2 ± 98.8 vs 95.9 ± 33.4 h (*p* = 0.003). At difference, full adherence to the ERAS components 91.7 ± 22.1 vs 92.2 ± 31.6 h (*p* = 0.8) was not associated with better recovery.

**Conclusions:**

Our results were encouraging to affirm that adherence to the ERAS program up to 75% could be considered satisfactory to get the goal. Our study could be considered a call to simplify the ERAS protocol facilitating its penetrance into clinical practice.

Enhanced recovery after surgery (ERAS) program is a perioperative protocol aimed at reducing surgical stress and improving postoperative functional recovery through the application of perioperative items in a multidisciplinary approach [1, 2].

Several reports demonstrated a strong association between the level of adherence to the protocol and improved clinical outcomes after surgery [3–5].

However, as known, it is difficult to obtain full adherence to the protocol into clinical practice and has not been identified so far the threshold beyond which improved functional results can be reached.

Furthermore, there is still a need for dedicated evaluation of individual ERAS components to identify which are associated with clear benefits [6].

An ad hoc study has been designed to prospectively evaluated how many and which items of the ERAS program are directly associated with functional recovery after colorectal surgery.

## Methods

### Study design

The ERCOLE (ERas and COLorectal Endoscopic surgery) study was designed as a cohort, prospective, multi-centre national study evaluating the association between adherence to ERAS items and clinical outcomes after minimally invasive colorectal surgery for cancer. The study obtained the approval and the endorsement of the SICE (Società Italiana di Chirurgia Endoscopica e Nuove Tecnologie – Italian Society of Endoscopic Surgery and New Technologies) and the Coordinator Center and Promoter of the study was the Department of Clinical Medicine and Surgery of the University of Naples “Federico II”.

The patients were recruited from September 2019 to September 2020.

Forty-five high referral Surgical Units for colorectal laparoscopic surgery joined the study. Data were collected using the official SICE website database.

The study was approved by the Institutional Review Board of all participating centres, and informed consent was obtained by all the included patients.

Each centre was asked not to change its own current practice: surgical approach and the pre- peri- and postoperative management (ERAS protocol compliance included).

All the ERAS items according to the last guidelines [2] were investigated, as well as the surgeons’ adherence to each ERAS item.

The primary endpoint was to associate the percentage of ERAS adherence to functional recovery after surgery when performing a minimally invasive treatment of colorectal cancer. Thus the study sample was stratified into quartiles of adherence.

Additionally, each ERAS item adherence and functional recovery has been separately analyzed.

Functional recovery after surgery was evaluated by a composite of postoperative outcomes. A successful recovery was defined as meeting all the following criteria: (1) complete mobilization, (2) stool passage, and (3) tolerance to a solid diet.

The secondary endpoint of the study was to validate safety of the ERAS programme evaluating complications’ occurrence according to Clavien-Dindo classification. Complications were defined as major if ≥ Clavien-Dindo Grade III.

Finally, the compliance of the Italian surgeons to each ERAS item and the reasons for non-compliance were reported.

### Population, inclusion/exclusion criteria, data extraction

Each centre was asked to enroll all consecutive cases observed during the study period according to inclusion and exclusion criteria.

Inclusion criteria were age > 18 years old, elective laparoscopic/ robotic colorectal cancer treatment.

Exclusion criteria were the inability to perform a minimally invasive approach and emergency surgery.

A section of the official SICE website allowed the online collection of the following data for each patient enrolled:

- Patient’s characteristics: gender, age, BMI, ASA score;
- Cancer’s characteristics: localization, size, stage;
- Surgical procedure: right colectomy, segmental transverse resection, segmental splenic flexure resection, left colectomy, sigmoidectomy, anterior rectal resection, abdominoperineal resection;
- Postoperative complications according to Clavien-Dindo classification;
- Adherence to ERAS protocol;
- Functional recovery after surgery: complete mobilization, stool passage, and tolerance to a solid diet.

### Statistical analysis

Statistical analysis was performed using the SPSS 26 system (SPSS Inc., Chicago, IL, USA). Continuous data were expressed as the means ± SDs, while categorical variables were expressed as percentages. Continuous variables were compared by an independent sample t-test. The Wilcoxon test for paired samples was employed as a non-parametric similar to the paired samples t-test used for continuous variables. Categorical data were analyzed by the chi-square test. Fisher’s exact test was employed when the minimum expected value was < 5.

Linear and logistic regression models were used to adjust for covariates (patients and cancers’ characteristics) and make predictions. Adopted covariates were gender, age, BMI, ASA Score, tumor localization, stage and surgical intervention.

All the results were presented as two-tailed values with statistical significance if *p* < 0.05.

## Results

The study included data of 1138 patients treated by minimally invasive surgery for colorectal cancer; 490 were females (43%) and 648 males (57%); the mean age was 70 ± 11.7 years; the mean BMI was 29.4 ± 3.8 kg/m^2^. A hundred and ten patients (9.7%) were judged ASA score I, 535 (47%) ASA score II, 445 cases (39.1%) ASA score III and 48 cases (4.2%) ASA score IV.

The tumour was located in 172 cases (15.1%) in the caecum, in 253 cases (22.2%) in the ascending colon, in 74 cases (6.5%) at the hepatic flexure, in 57 cases (5%) in the transverse colon, in 41 cases (3.6%) at the splenic flexure, in 64 cases (5.6%) in the descending colon, in 174 cases (15.4%) in the sigmoid tract and in 303 (26.6%) in the rectum. The mean tumour size was 7.4 ± 2.9 cm. A hundred and thirty-seven patients (12%) were AJCC stage I, 208 (18.3%) AJCC stage II, 353 (31%) AJCC stage III and 440 (38.7%) AJCC stage IV.

A right colectomy was performed in 519 cases (45.6%), a left colectomy in 270 cases (23.7%), a splenic flexure resection in 26 cases (2.3%), a segmental transverse resection in 20 cases (1.7%), a rectal resection in 279 cases (24.5%) and an abdominoperineal resection in 24 cases (2.1%).

Complications after surgery occurred in 364 cases (32%). According to Dindo-Clavien classification, 139 patients (12.2%) had a complication classified as grade I, 128 cases (11.3%) as grade II, 72 cases (6.3%) as grade III and 25 cases (2.2%) as grade IV.

Functional recovery after surgery, as described by our composite outcome, was 92.1 ± 30.6 h. Mobilization was reported to be 25.2 ± 22.7 h, stool passage 68.4 ± 32.7 h, tolerance to solid diet 12.4 ± 8.6 h (Table 1).

**Table 1:** Non-adherence to the ERAS items and reasons

ERAS	NO COMPLIANCE	Reasons		
		Habits	Surgeons’ disagreement	Organization
Counselling	21%	8.4%	35.6%	56%
Smoking and alcohol cessation	42%	5.4%	43.8%	50.8%
Physical Prehabilitation	63%	3.1%	30.1%	66.8%
Nutritional status screening	26.5%	3.3%	38.1%	58.6%
Management of anaemia	1.3%	33%	0%	67%
PONV prophylaxis	23.1%	0.4%	95%	4.6%
No premedication	5.3%	1.7%	6.7%	91.6%
Antibiotic prophylaxis	2%	0%	0%	100%
No bowel preparation	20%	0%	0%	100%
Preoperative fluid management	2.1%	1%	0%	99%
Preoperative fasting	29.4%	2%	0%	98%
Oral carbohydrates load	51.9%	2%	0%	98%
Standard anaesthetic protocol	1.5%	5.9%	0%	94.1%
Intraoperative fluid management	1.6%	5.6%	0%	94.4%
Normothermia	7.6%	0%	0%	100%
No drain	48.2%	4.6%	0%	95.4%
No nasogastric tube	10.4%	0%	6.8%	93.2%
Multimodal analgesia	14.7%	0%	1%	99%
Antithrombotic prophylaxis	1.7%	0%	0%	100%
Postoperative fluid management	3.3%	0%	0%	100%
Bladder catheter	1.8%	0%	38%	62%
Prevention of ileus (nasogastric tube, no fluid overload)	3.3%	0%	5.3%	94.7%
Prevention of ileus (chewingum/alvimopan)	72.1%	0%	1.8%	98.2%
Nutritional prehabilitation and immunonutrition	9%	0%	48%	52%
Early oral feeding	30%	0%	5%	95%
Early mobilization	19.2%	0%	11.5%	88.5%

### Adherence to the ERAS program

Full adherence to the ERAS protocol has been registered only in 101 out of 1138 patients (8.9%), with 181 (15.9%) adhering to the preoperative part, 555 (48.8%) to the perioperative part and (18.4%) to the postoperative part.

Adherence to the ERAS program has been registered to be > 75% in 736 (64.7%) and > 50% in 1127 (99%). Adherence to each item and reasons for non-compliance are shown in Table 2.

**Table 2:** ERAS items not correlated with better functional recovery after surgery

Item	Compliance		Composite outcomes (hours)		*P*
	Yes	No	Yes	No	
Physical prehabilitation	37%	63%	89.67 ± 25.66	93.04 ± 32.57	0.1
Nutritional status screening	73.5%	26.5%	92.61 ± 32.5	90.75 ± 25.79	0.4
Preoperative administration of complex carbohydrates	48.1%	51.9%	90.36 ± 30.07	93.75 ± 31.51	0.1
No premedication	94.7%	5.3%	91.93 ± 30.9	95.6 ± 30.13	0.4
Intraoperative normothermia	92.4%	7.6%	92.39 ± 31.25	88.82 ± 25.65	0.3
Multimodal analgesia	85.3%	14.7%	92.76 ± 31.84	88.38 ± 24.15	0.1
Prevention of ileus with chewing gum or alvimopan	96.7%	3.3%	92.29 ± 29.06	92.05 ± 31.55	0.9
Early oral feeding	70%	30%	91.39 ± 31.25	93.82 ± 29.93	0.2
Early mobilization	80.8%	19.2%	92 ± 32.21	92.59 ± 24.42	0.8

### Adherence to the ERAS and functional outcomes

Successful recovery after surgery, as defined by the composite outcome (complete mobilization, stool passage and tolerance to solid diet), was associated with compliance to the ERAS protocol.

We found that adherence to > 75% was associated with a better functional recovery with 90.2 ± 29.15 vs 95.9 ± 33.4 h (*p* = 0.003).

**Fig. 1:**
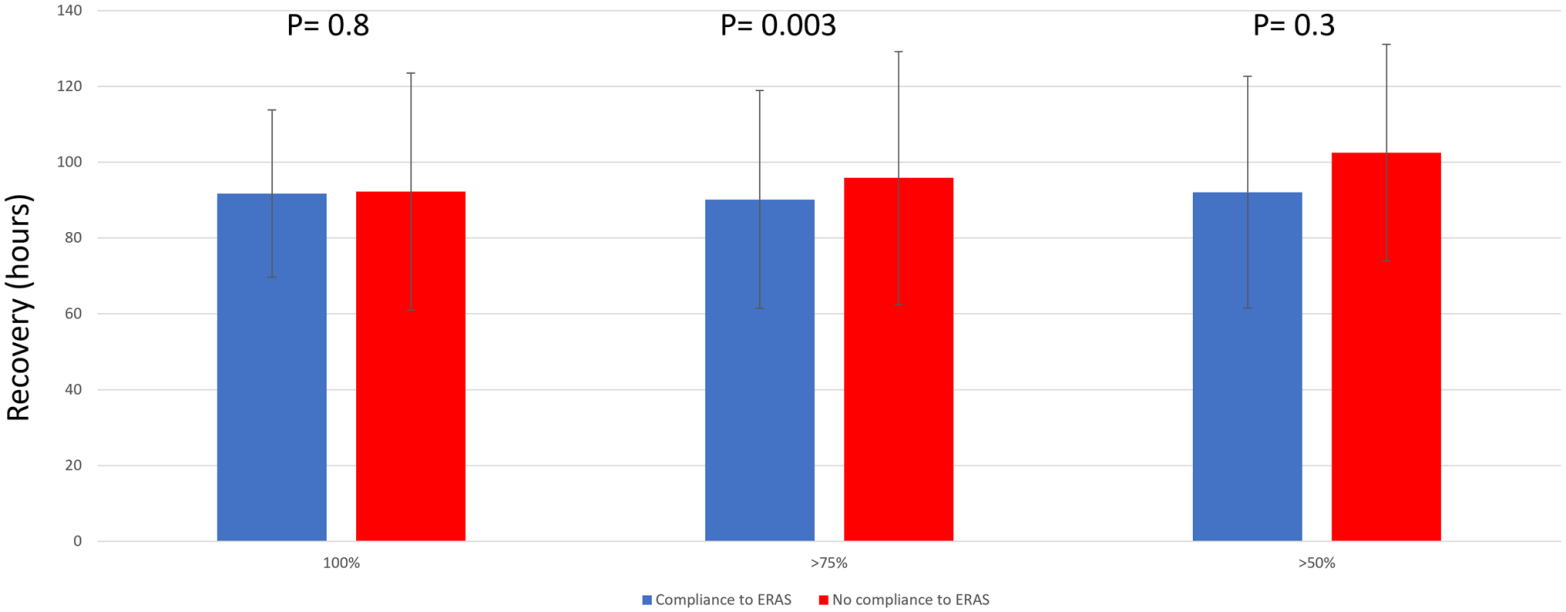
Functional Recovery and Compliance to the ERAS

At difference, full adherence to the ERAS components 91.7 ± 22.1 vs 92.2 ± 31.6 h (*p* = 0.8) was not associated with a better functional recovery. Similarly, the adherence to > 50% with 92.1 ± 30.6 vs 102.5 ± 28.6 h (*p* = 0.3) was not associated to better outcomes even if results were affected by excessive numerical gab between the groups (only 1% of adherence < 50%). Association between ERAS components and functional recovery is shown in Fig. 1.

The analysis of association between each ERAS item adherence and recovery after surgery showed that few items were not associated with better recovery (Table 2). In details, physical prehabilitation with 89.7 ± 25.7 vs 93.0 ± 32.6 h (*p* = 0.1), nutritional status screening with 92.6 ± 32.5 vs 90.7 ± 25.8 h (*p* = 0.4), preoperative administration of complex carbohydrates with 90.7 ± 30.1 vs 93.7 ± 31.5 h (*p* = 0.1), no premedication with 91.9 ± 30.9 vs 95.6 ± 30.1 h (*p* = 0.4), intraoperative normothermia with 92.4 ± 31.2 vs 88.8 ± 25.6 h (*p* = 0.3), multimodal analgesia with 92.76 ± 31.8 vs 88.4 ± 24.1 h (*p* = 0.1), prevention of ileus with chewing gum or alvimopan with 92.3 ± 29.1 vs 92.0 ± 31.5 h (*p* = 0.9), early oral feeding with 91.4 ± 31.2 vs 93.8 2 ± 29.9 h (*p* = 0.2), early mobilization with 92.0 ± 32.2 vs 92.6 ± 24.4 h (*p* = 0.8) did not influence the recovery of the patient.

Multivariate analyses excluded any bias related to patients’, cancers’ characteristics and surgical procedure. In details, multivariate analyses showed no bias related to gender (*p* = 0.225), age (*p* = 0.159), BMI (*p* = 0.571), ASA Score (*p* = 0.064), tumor size (*p* = 0.808).

### Adherence to the ERAS and complications

**Fig. 2:**
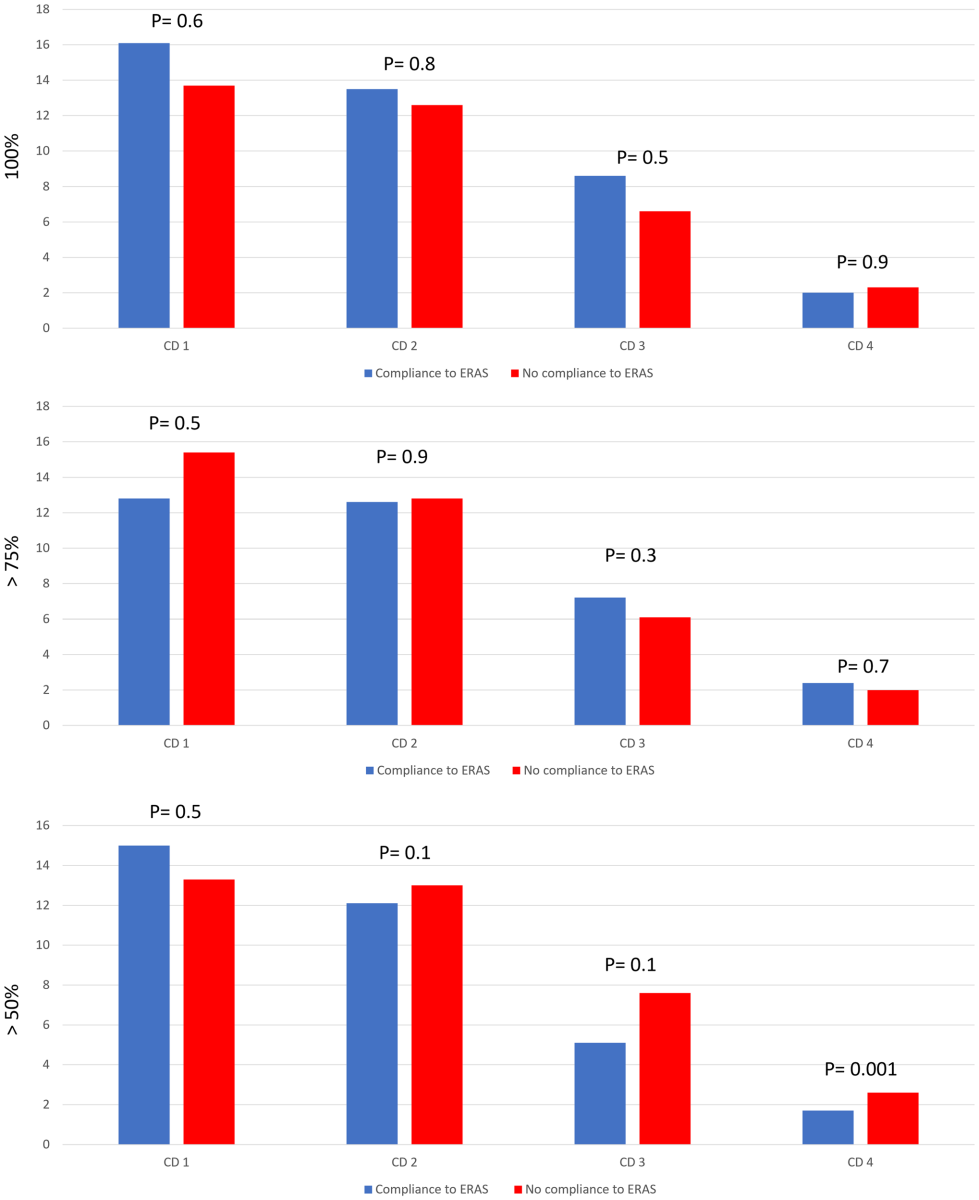
Complications’ occurrence and Compliance to the ERAS

The association between compliance to the ERAS components and postoperative complications is shown in Fig. 2. The compliance to the ERAS was not associated with the occurrence of complications after surgery.

The association between adherence to each ERAS item and postoperative complications, classified according to the Clavien-Dindo showed that few items were associated with the occurrence of the complications and all were minor complications. In details, Clavien-Dindo grade I complications were associated with adherence to PONV prophylaxis (*p* = 0.02), to preoperative fluid management (p = 0.003), to early nasogastric tube removal (*p* = 0.024), to early bladder catheter removal (*p* = 0.001), to the prevention of ileus with use of a nasogastric tube and no fluid overload (*p* = 0.04), to nutritional habilitation and immunonutrition (*p* = 0.02) and to early mobilization (*p* = 0.004). Clavien-Dindo grade II complications were associated with adherence to no premedication (*p* = 0.03) and to no bowel preparation (*p* = 0.02).

Specifically, the adherence to all of the above mentioned ERAS items was associated with a lower rate of postoperative complications.

Multivariate analyses excluded any bias related to patients’, cancers’ characteristics and surgical procedure. In details, multivariate analyses showed no bias related to gender (*p* = 0.097), age (*p* = 0.113), BMI (*p* = 0.672), ASA Score (*p* = 0.088), tumor size (*p* = 0.569).

## Discussion

Enhanced recovery after surgery (ERAS) represents a multimodal evidence-based approach to optimize the perioperative management of patients [7–10]. The program relies on a series of evidence-based items related to pre-, intra- and postoperative care [11, 12].

Multiple meta-analyses on ERAS have shown a significant reduction in morbidity and length of stay after colorectal surgery [13–15]. However, there is high variability in the program implementation outside clinical trials [16–18], as the involvement of several specialists makes the program difficult to be applied in all of its parts, explaining the great variation in adherence rates to program items [11, 19–21].

Additionally, from a clinical point of view, many surgeons relied only on a limited number of elements for personal habits and beliefs. It is important to highlight that Kehlet’s original description of the ERAS protocol comprised 26 different elements, and even if there is evidence to suggest that increased overall compliance improves clinical outcome [22–24], the impact of individual protocol elements on the outcome is still unknown. Other authors suggested a simplified ERAS protocol known as RAPID (remove, ambulate, postoperative analgesia, introducing diet) [25]. This protocol suggests the removal of nasogastric tubes at the end of the intervention, early mobilization, early oral fluids, and early diet reintroduction, reduction of analgesia with opioids, removal of a urinary catheter, and stop of intravenous fluids at postoperative day 3.

The RAPID protocol could be considered an answer against ERAS protocol, even if it could be also considered the idea of some surgeons that not all items of the ERAS are very useful to improve results [26]. Up to now, from current evidence, we know that pooled compliance to the ERAS protocol was 69, 72 and 53% with pre- peri- and postoperative ERAS protocol [6], even if it is important to highlight that the results have been registered by surgeons committed, at least, to the implementation of the protocol. On a clinical point of view these percentages, maybe, could be considered too high if compared with all hospital’s units worldwide.

Thus, we have designed an ad hoc study to prospectively associate clinical outcomes with adherence to ERAS elements. We decided to include only minimally invasive procedures for colorectal cancer treatment in high volume centres for colorectal surgery to homogenize the results. Minimally invasive surgery is considered the ideal treatment of colorectal cancer, and the link with a better recovery could be considered strengthened. Thus we have excluded open surgery thinking that it could be considered a bias evaluating our study the functional results as primary outcome.

We aimed to give a snapshot of current adherence to ERAS protocol in Italy inviting and including all surgeons regardless their full adherence to the ERAS protocol.

This report on the compliance of Italian surgeons to the ERAS Program shows a very low rate of full adherence to the ERAS program in clinical practice. On the other hand, high adherence to the program—> 75%—was widely obtained, regardless to the adoption of an institutional ERAS protocol.

In detail, fully adherence (100%) to the ERAS program has been registered only in 9% while it has been reported > 75% in 64.7%; almost in all (99%) an adherence > 50% has been identified.

It was worth mentioning that improving functional results have been obtained with an adherence > 75%.

Thus the cultural barrier to the implementation of the ERAS program should be abandoned. Again, our results confirmed the advantages of an ERAS strategy in minimally invasive colorectal surgery. However, a point that could substantiate the value of the ERAS program was to give an answer to the provocative question by critics: what are the advantages of the ERAS program? And above all, how many and which items should be effectively considered important to improve the results?

Even if it should be stated that functional recovery after surgery was promoted, we confirm that a full adherence to the ERAS program could be considered useless to improve recovery after surgery. Both the occurrence of complications and functional recovery after surgery were not improved by complete adherence to all ERAS items.

It is worth mentioning that an adherence > 75% has to be considered satisfactory to obtain the advantages of the ERAS program, especially in terms of faster recovery. However, we have to take into consideration that an adherence > 75%, although significant in terms of statistical value, is associated with a not clinically significant difference (about 5 h).

Nevertheless, our results were encouraging to affirm that adherence to the ERAS program up to 75% could be considered satisfactory to get the goal. On the other hand, we have to take into consideration that an adherence > 75% was.

Additionally we have tried to identify which items are linked to better recovery outcomes.

Messenger et al. [6], in a systematic review, already aimed to identify the individual ERAS elements and protocol compliance that were linked with outcomes. However, although 14 studies reported individual compliance, meta-analysis beyond pooled compliance was not possible due to wide study heterogeneity in the research question, design, endpoints and large differences in the number and nature of individual elements included in ERAS programs and incomplete reporting.

In the setting of the ERAS program implementation, it could be interesting to report which ERAS items are not directly related with an enhanced recovery after surgery.

In a retrospective analysis on 733 patients Vignali et al. [3] demonstrated that only non-compliance with the intra-operative balanced fluid therapy, failure to early removal of the urinary catheter, to discontinue intravenous fluid and to early mobilization were independently associated with ERAS failure.

On the contrary, Catarci et al. [11] demonstrated on 196 consecutive colorectal resection that the adherence to all ERAS items was associated with a significant dose–effect curve for overall and major morbidity rate, anastomotic leakage and length of stay.

In this setting, our results are in contradiction with other studies.

In fact, our results demonstrated that physical prehabilitation, nutritional status screening, preoperative administration of complex carbohydrates, no premedication, intraoperative normothermia, multimodal analgesia, prevention of ileus with chewing gum or alvimopan, early oral feeding and early mobilization were not associated with an earlier postoperative recovery.

Additionally, some of these points were very difficult to be included in clinical practice, and if not associated with clear advantages, we propose that they could be abandoned by a revised ERAS program. On the other hand, a revised ERAS program should include all the items significantly impacting the functional results, i.e., preoperative counselling, preoperative physical optimization, management of the anaemia, prevention of nausea and vomiting, antimicrobical prophylaxis, no bowel preparation, intraoperative euvolemia, no adoption of abdominal drains, early nasogastric tube and urinary drainage removal, postoperative analgesia, antithrombotic prophylaxis.

However, despite these results, major limitations of the current report should be stated. First, this study is an observational report, thus affected by several intrinsic biases. Second, this is a multicentric study, thus affected by extreme heterogeneity among the centers. Third, centers included were not selected by adherence to an institutional ERAS program, thus adherence to each items could be only related by surgeons’ decision.

However, the limitations could also be considered the strength of our study. It is a snapshot of current clinical practice in Italy among surgeons addicted to minimally invasive surgery. In Italy there is not a Standardized National Protocol of Adherence to the ERAS program neither national surgical society consensus or recommendation to guide surgeons in the implementation of adherence to the ERAS. This study could be considered a call to standardize the adherence to the ERAS giving society recommendation, also evaluating to simplify the protocol facilitating its penetrance into clinical practice.

After that, ad hoc randomized controlled trials specifically addressing the impact of ERAS items’ adherence on clinical outcomes should be performed to obtain definitive conclusion.
