# Correction: ERas and COLorectal endoscopic surgery: an Italian society for endoscopic surgery and new technologies (SICE) national report

**DOI:** 10.1007/s00464-022-09344-1

**Published:** 2022-05-24

**Authors:** Marco Milone, Ugo Elmore, Michele Manigrasso, Monica Ortenzi, Emanuele Botteri, Alberto Arezzo, Gianfranco Silecchia, Mario Guerrieri, Giovanni Domenico De Palma, Ferdinando Agresta, ERCOLE Study Group, Ferdinando Agresta, Francesco Pizza, Dario D’Antonio, Francesco Amalfitano, Francesco Selvaggi, Guido Sciaudone, Lucio Selvaggi, Daniela Prando, Fabio Cavallo, Mario Guerrieri, Monica Ortenzi, Giovanni Lezoche, Diego Cuccurullo, Ernesto Tartaglia, Carlo Sagnelli, Andrea Coratti, Angela Tribuzi, Michele Di Marino, Gabriele Anania, Cristina Bombardini, Mauro Pietro Zago, Fulvio Tagliabue, Morena Burati, Salomone Di Saverio, Samuele Colombo, Sara El Adla, Maurizio De Luca, Monica Zese, Dario Parini, Paolo Prosperi, Giovanni Alemanno, Jacopo Martellucci, Stefano Olmi, Alberto Oldani, Matteo Uccelli, Dario Bono, Donatella Scaglione, Roberto Saracco, Mauro Podda, Adolfo Pisanu, Valentina Murzi, Antonino Agrusa, Salvatore Buscemi, Irnerio Angelo Muttillo, Biagio Picardi, Edoardo Maria Muttillo, Leonardo Solaini, Davide Cavaliere, Giorgio Ercolani, Francesco Corcione, Roberto Peltrini, Umberto Bracale, Andrea Lucchi, Laura Vittori, Michele Grassia, Alberto Porcu, Teresa Perra, Claudio Feo, Pierluigi Angelini, Domenico Izzo, Luigi Ricciardelli, Mario Trompetto, Gaetano Gallo, Alberto Realis Luc, Andrea Muratore, Marcello Calabrò, Bruno Cuzzola, Andrea Barberis, Federico Costanzo, Giulio Angelini, Graziano Ceccarelli, Fabio Rondelli, Michele De Rosa, Elisa Cassinotti, Luigi Boni, Ludovica Baldari, Paolo Pietro Bianchi, Giampaolo Formisano, Giuseppe Giuliani, Andrea Alessandro Pisani Ceretti, Nicolò Maria Mariani, Marco Giovenzana, Roberto Farfaglia, Paolo Marcianò, Valeria Arizzi, Micaela Piccoli, Francesca Pecchini, Gianmaria Casoni Pattacini, Emanuele Botteri, Nereo Vettoretto, Claudio Guarnieri, Letizia Laface, Emmanuele Abate, Massimiliano Casati, Carlo Feo, Nicolò Fabri, Antonio Pesce, Piero Maida, Giampaolo Marte, Roberta Abete, Lorenzo Casali, Alessandro Marchignoli, Matteo Dall’Aglio, Stefano Scabini, Davide Pertile, Alessandra Aprile, Jacopo Andreuccetti, Alberto Di Leo, Lorenzo Crepaz, Francesco Maione, Sara Vertaldi, Alessia Chini, Riccardo Rosati, Francesco Puccetti, Giulia Maggi, Andrea Cossu, Alberto Sartori, Maurizio De Luca, Giacomo Piatto, Nicola Perrotta, Marta Celiento, Marco Scorzelli, Vincenzo Pilone, Salvatore Tramontano, Pietro Calabrese, Raffaele Sechi, Nicola Cillara, Giaime Putzu, Michele Guido Podda, Mauro Montuori, Enrico Pinotti, Giuseppe Sica, Marzia Franceschilli, Bruno Sensi, Maurizio Degiuli, Rossella Reddavid, Lucia Puca, Marco Farsi, Alessio Minuzzo, Elena Gia, Gian Luca Baiocchi, Valerio Ranieri, Andrea Celotti, Francesco Bianco, Sebastiano Grassia, Alessandra Novi

**Affiliations:** 1 Department of Clinical Medicine and Surgery, Federico II” University of Naples, via Pansini 5, 80131 Naples, Italy; 2 Division of Gastrointestinal Surgery, San Raffaele Scientific Institute, Milan, Italy; 3 Department of Advanced Biomedical Sciences, “Federico II” University of Naples, via Pansini 5, Naples, Italy; 4 Department of General Surgery, Università Politecnica Delle Marche, Piazza Roma 22, 60121 Ancona, Italy; 5 General Surgery, ASST Spedali Civili Di Brescia, Montichiari, Italy; 6 Department of Surgical Sciences, University of Torino, Turin, Italy; 7 Department of Medical Surgical Science and Biotechnologies, Faculty Pharmacy and Medicine, Sapienza University of Rome, Rome, Italy; 8 Department of General Surgery, Department of General Surgery, Ulss2 Marca Trevigiana, Vittorio Veneto, TV Italy

## Correction to: Surg Endosc (2022) 10.1007/s00464-022-09212-y

This article was updated to correct Nicolò Fabbri's name in the listing of the ERCOLE Study Group (in Acknowledgments).

